# Neurological Researches

**Published:** 1838-07

**Authors:** 


Art. XV.—*Neurologische Beobachlungen.* Von Dr. F. H. Bidder.
*Neurological Researches.* By Dr. F. H. Bidder, Prosector in the Anatomical School of Dorpat.—*Dorpat*, 1836. 4to. pp. 58.

Dr. Bidder's researches lead to the conclusion that the nerves of the tentorium arise from the fourth nerve, and not from the first division of the fifth. This agrees with Arnold's *first* account of these nerves. They arise from the fourth nerve soon after it has entered the dura mater; then run in the groove between the posterior clinoid process and petrous bone. Sometimes these nerves may be traced continuous with those of the sympathetic which ascend to join the fourth: Dr. Bidder considers the nerves of the tentorium as sympathetic nerves.

The muscles of the soft palate which are brought into action in swallowing, vomiting, and in the commencement of sneezing, receive branches not only from the n. pterygo-palatinus of the second division of the fifth, but also from the glosso-pharyngeus. Dr. Bidder considers the former not only a sensitive, but also a motor nerve; and as the first two divisions of the fifth are merely sensitive, he derives the motor portion from the communication of the facial with the ganglion spheno-palatinum, by the nervus petrosus superficialis, which he says arises from the central part of the facial, and which is contrary to former investigations.

Dr. Bidder describes a nervus petrosus superficialis tertius: it proceeds from the plexus which accompanies the arteria meningea media; lies first in the base of the skull, nearly covered by dura mater, or rather between the laminæ of this membrane: soon, however, it enters an opening on the anterior surface of the petrous bone before and under the aditus canalis Fallopii, and passing to the facial, joins it behind the knee or in the swelling of the knee. Once he saw it come from the nervus petrosus superficialis minor, which divided into three branches,—one to the anastomosis of Jacobson, one to the knee of the facial, and the third joined the facial behind the knee. The nerves which accompany the arteria meningea media send twigs to the dura mater.

Dr. Bidder observes that he has never found the n. petrosus sup. minor lying free on the petrous bone, but always covered by bone; it does not pass through the foramen ovale, but through the sphenoid and petrous bones, and appears afterwards on the surface of the latter.

